# Biodiversity of zooplankton (Rotifera, Cladocera and Copepoda) in the tributaries of Cheboksary Reservoir (Middle Volga, Russia)

**DOI:** 10.3897/BDJ.12.e116330

**Published:** 2024-02-13

**Authors:** Dmitriy Gavrilko, Vyacheslav Zhikharev, Tatyana Zolotareva, Ivan Kudrin, Basil Yakimov, Aleksandra Erlashova

**Affiliations:** 1 National Research Lobachevsky State University of Nizhny Novgorod, Nizhny Novgorod, Russia National Research Lobachevsky State University of Nizhny Novgorod Nizhny Novgorod Russia

**Keywords:** species richness, occurrence, abundance, centre of European Russia

## Abstract

**Background:**

Freshwater zooplankton is an important component of the ecological communities of inland water bodies. It acts as an important part of the food web and participates in the self-purification processes of aquatic ecosystems. To study the abundance and distribution of species, a sampling event dataset was compiled and then published through GBIF. The aim of the work was to describe the current zooplankton fauna (Rotifera, Cladocera and Copepoda) and its abundance, based on a recently published dataset. The research was conducted from 2015 to 2022. Zooplankton samples were collected by vertical towing a plankton net (70 μm mesh) from the bottom to the water surface or by filtering through a net, the water being collected with a measuring bucket. The samples were concentrated to 100 ml and fixed with a final concentration of 4% formalin solution. For each sampling event, the coordinates of the location, number of individuals and date were recorded.

**New information:**

The dataset contains information on 259 taxа, including 257 species and subspecies of zooplankton from 36 families found in the tributaries of the Cheboksary Reservoir. The families Chydoridae (35 species), Brachionidae (31) and Cyclopidae (27) were the most species-rich. Four invasive species were found: *Kellicottiabostoniensis* (Rousselet, 1908), *Acanthocyclopsamericanus* (Marsh, 1893), *Ilyocryptusspinifer* Herrick, 1882 and *Thermocyclopstaihokuensis* Harada, 1931.

## Introduction

Freshwater zooplankton is an important component of ecological communities of inland water bodies. It includes invertebrates from different systematic groups, the main ones being rotifers, cladocerans and copepods. Acting as an important part of the food web, zooplankton participates in the self-purification processes of aquatic ecosystems and is a food base for fish and other invertebrates (Moss 1988, Wetzel 2001, Bruce et al. 2006, Błędzki and Rybak 2016, Sharma 2020).

Rivers are the most widespread type of water body in the world. They have rich faunistic diversity and their estuaries act as refuges for zooplankton (Krylov et al. 2010, Mukhortova et al. 2015, Zhikharev et al. 2023a, Zhikharev et al. 2023b). River ecotones formed in estuaries maintain high biodiversity, bioproduction and nutrient transformation (Ward and Wiens 2001, Ward et al. 2002).

In recent decades, the increasing anthropogenic impact on aquatic ecosystems has resulted in accelerated pollution, eutrophication and, as a consequence, changes in the biodiversity of aquatic communities (Loreau et al. 2001, Naeem et al. 2012, Zhikharev et al. 2023b). Planktonic crustaceans and rotifers are sensitive to eutrophication of aquatic ecosystems and could change species diversity and spatial distribution of communities (Shurganova et al. 2018, Afonina and Tashlykova 2020, Krupa et al. 2020, Liang et al. 2020, Waite et al. 2020, Zhikharev et al. 2021). The invasion of alien species into water bodies poses a particular threat to biodiversity. By invading ecosystems, invasive species can displace native species and reduce local biodiversity (Havel et al. 1995, Swaffar and O'Brien 1996, Zanata et al. 2003, Mergeay et al. 2004, Strecker and Arnott 2008, Wittmann et al. 2013, Walsh et al. 2016). Often rivers act as transit corridors for the distribution of zooplanktonic invasive species (Lazareva and Zhdanova 2014, Lazareva 2019, Lazareva et al. 2022) and their estuaries could be acclimatisation habitats (Zhikharev et al. 2023a). Knowledge about the findings of alien species in new habitats is necessary for monitoring the process of their dispersal.

A large number of works have been devoted to the study of zooplankton in the Middle Volga Basin. However, there are very few collections with records of species abundance (Mukhortova et al. 2021). There is a certain gap in documenting data on zooplankton species occurrence and abundance in the Middle Volga Basin. The use of free platforms for documentation and the creation of a dataset that can be accessed by all users allow biodiversity assessment and reproducible analyses (Mazurov et al. 2022).

## General description

### Purpose

The aim of this paper was to describe the current zooplankton fauna and abundance of the tributaries of the Cheboksary Reservoir, based on a recently published dataset (Gavrilko et al. 2023).

## Project description

### Title

Biodiversity of Zooplankton (Rotifera, Cladocera and Copepoda) in the Tributaries of Cheboksary Reservoir (Middle Volga, Russia)

### Personnel

Dmitriy Gavrilko, Vyacheslav Zhikharev, Tatyana Zolotareva, Ivan Kudrin, Basil Yakimov, Aleksandra Erlashova

### Study area description

**Figure 1.**
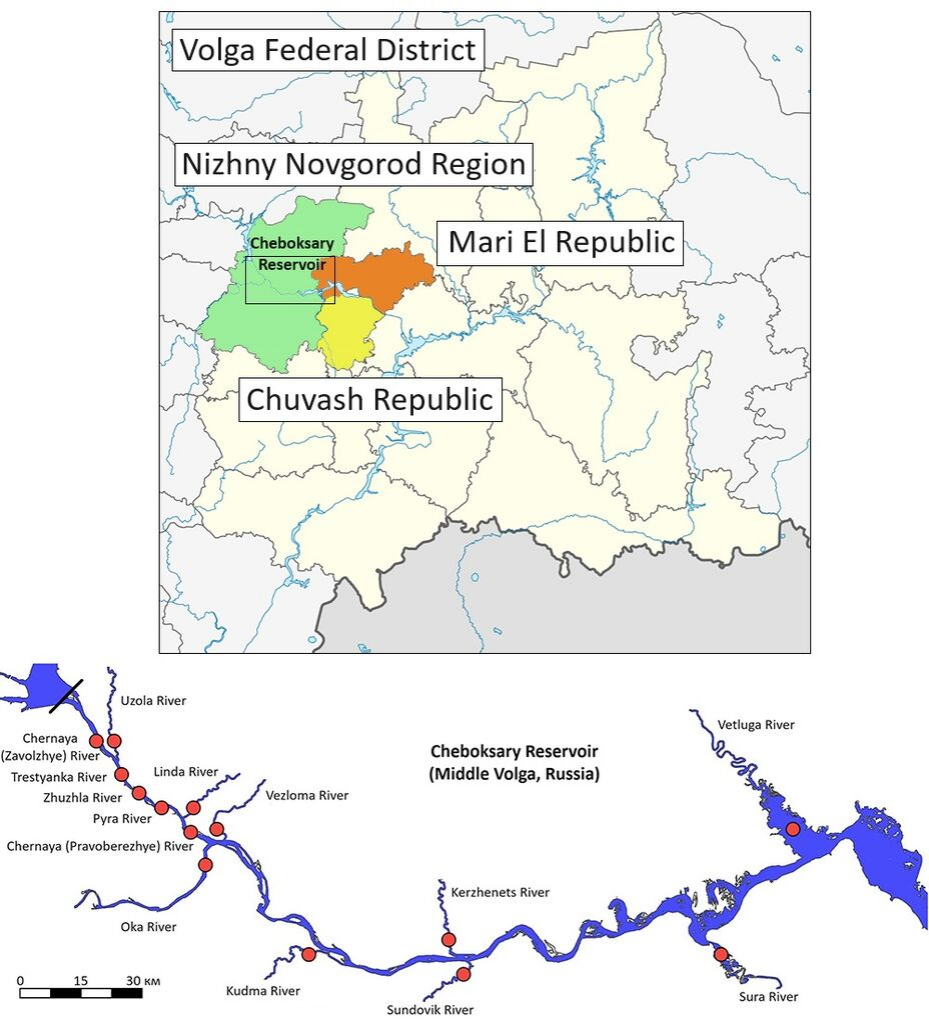
Location map of the studied tributaries of the Cheboksary Reservoir (circles indicate tributaries where samples were collected).

The studies were conducted in tributaries of the Cheboksary Reservoir (Nizhny Novgorod Oblast and the Republic of Mari El). Hydrobiological data were obtained and published between 2015 and 2022 from 14 rivers: Vetluga, Sura, Kerzhenets, Sundovik, Kudma, Vezloma, Oka, Linda, Pyra, Trestyanka, Chernaya (Zavolzhye), Zhuzhla, Chernaya (Pravoberezhye) and Uzola (Fig. 1).

## Sampling methods

### Study extent

The presented dataset on the taxonomic composition and abundance of zooplankton in tributaries of the Cheboksary Reservoir is based on the original materials (samples) of the authors. The species list includes native species and naturalised species, including invasive species. The dataset represents mainly native species (98.4%), with invasive species accounting for no more than 1.6%. Studies were carried out in tributaries of the Cheboksary Reservoir (Nizhny Novgorod Region and the Republic of Mari El, European Russia). Hydrobiological data were obtained and published from 2015 to 2022 from 14 rivers: Vetluga, Sura, Kerzhenets, Sundovik, Kudma, Vezloma, Oka, Linda, Pyra, Trestyanka, Chernaya (Zavolzhye), Zhuzhla, Chernaya (Pravoberezhye) and Uzola.

### Sampling description

The identification of the species composition of zooplankton was performed in 2015–2022. When specifying the taxonomic affiliation of zooplankton, we used proper manuals and guides (Kutikova 1970, Alekseev and Tsalolikhin 2010, Błędzki and Rybak 2016, Rogers and Thorp 2019, Korovchinsky et al. 2021). Species lists were checked against checklists (Segers 2007, Kotov et al. 2013) as well as the World of Copepods database in the Catalogue of Life (Walter and Boxshall 2023). The identification of invasive species was carried out using the work of a number of researchers (De Paggi 2002, Lazareva and Zhdanova 2014) for *Kellicottiabostoniensis*, (Monchenko 2008) for *Thermocyclopstaihokuensis*, (Korovchinsky et al. 2021) for *Ilyocryptusspinifer* and (Alekseev 2021) for *Acanthocyclopsamericanus*.

### Quality control

All samples were identified by the researchers working at the Lobachevsky State University of Nizhnii Novgorod and stored in the scientific collection of the university. The reliability of the taxonomic definitions was confirmed by taxonomists of the A.N. Severtsov Institute of Ecology and Evolution, Russian Academy of Sciences (Korovchinsky et al. 2021). The taxonomic nomenclature is given in accordance with the taxonomic system of GBIF Backbone Taxonomy (GBIF Secretariat 2021). In order to publish the dataset on the GBIF network, the records have been adjusted according to the Darwin Core specifications (Wieczorek et al. 2012).

### Step description

Zooplankton samples were collected by vertical towing a plankton net (70 μm mesh) from the bottom to the water surface or by filtering through a net, the water being collected with a measuring bucket. The samples were concentrated to 100 ml and fixed to a final concentration of 4% formalin solution (Mordukhay-Boltovskoy 1975). Zooplankton specimens were examined using a Zeiss Stemi 2000C stereomicroscope (Carl Zeiss Microscopy, Germany) and a detailed morphological analysis was performed using an Olympus CX43 light microscope (Olympus Crp., Japan). Studies were conducted mostly in the lower reaches and estuaries of the rivers. A total of 200 zooplankton samples were processed. After processing, all samples were stored in the authors' personal collections for further detailed taxonomic and morphological studies.

## Geographic coverage

### Description

The Cheboksary Reservoir is the fifth in the cascade of Volga reservoirs and is located in the central part of the East European Plain. This territory is part of the temperate continental climate. There are great seasonal differences in the duration of the daylight hours and the sun's height above the horizon in the temperate zone. There is a rapid decrease of solar radiation to the north in winter. Maximum daily sums of solar radiation are observed during May–July at the highest altitudes of the sun and maximum day length. In average annual output, the inflow of total solar radiation in this climate is almost 2 times less than in tropical climates. Cloudiness reduces the inflow of total solar radiation by an average of 40 percent. Radiation balance is the main factor in heating and cooling the air. It also regulates moisture evaporation from the surface. The annual course of turbulent heat transfer is characterised by a summer maximum that increases with increasing dryness. In winter, the turbulent heat flux is directed from the atmosphere to the Earth's surface, but its absolute values are smaller than in summer. Annual precipitation on the plain territory in this climate varies from 300 to 800 mm (Sorokina and Gushchina 2006).

The Cheboksary Reservoir is 341 km long, with an average depth of 4.7 m and a maximum depth of 21 m. It has the highest flow capacity and water exchange coefficient not only amongst the reservoirs of the Middle Volga, but also of the entire Volga cascade.

From Gorodets to the mouth of the Oka River, the Reservoir is located on the Balakhna Plain and has relatively symmetrical banks. Below the mouth of the Oka River, the right bank is high and steep (up to 100 m high) and the left bank is low. The largest tributary of the Reservoir is the Oka River (Mineeva et al. 2021). The Reservoir is located in the most densely populated industrial regions of European Russia and experiences a serious anthropogenic load (Mineeva 2004, Sigareva et al. 2005). The main hydrological characteristics of the studied tributaries of the Cheboksary Reservoir are presented in Table 1.

## Taxonomic coverage

### Description

The dataset provides information on 259 taxа, including 257 species and subspecies of zooplankton, as well as two genera *Bythotrephes* Leydig, 1860 and *Notommata* Ehrenberg, 1830 (rotifers – 143, cladocerans – 80, copepods – 34) from 36 families found in tributaries of the Cheboksary Reservoir (Table 2). The families Chydoridae (35 species), Brachionidae (31) and Cyclopidae (27) were the largest in terms of species richness.

Most rotifers of the order Bdelloidea could not be identified as species in the fixed material, so they were recorded as order Bdelloidea.

To compare our data with the species richness of zooplankton from the basins of other Volga reservoirs: small rivers of the Upper Volga basin – 157 species (Krylov 2005), Rybinsk Reservoir – more than 350 species (Lazareva 2010), Kuibyshev Reservoir Basin – 111 species (Mukhortova et al. 2021). Significant differences in the number of species from different regions are related to several problems: different years of research, different types of water bodies and different study sites. Most researchers study pelagic zooplankton without sampling littoral macrophyte thickets. However, in rivers, the greatest species richness of zooplankton is concentrated in macrophyte thickets (Gavrilko et al. 2019, Gavrilko et al. 2020).

Findings of alien zooplankton species are of great importance for studying the processes of dispersal of invasive species in aquatic ecosystems. We found four alien species in the samples: two transcontinental invaders, *Kellicottiabostoniensis* (Rousselet, 1908) and *Acanthocyclopsamericanus* (Marsh, 1893) and two tropical invaders, *Ilyocryptusspinifer* Herrick, 1882 and *Thermocyclopstaihokuensis* Harada, 1931. The rotifer *K.bostoniensis* and the copepoda *A.americanus* had the highest occurrence frequency. The cladocera *I.spinifer*, originally found in Europe in the Vetluga River (Zhikharev et al. 2020), was first found in the Kerzhenets River in a thicket of *Stratiotesaloides* L., 1753. In contrast, the copepoda *T.taihokuensis* was found exclusively in the riparian zone of the Vetluga River. Recently, this species has been invading new habitats in the Volga Basin (Lazareva 2022).

## Temporal coverage

### Notes

The presented dataset contains information on the occurrence of zooplankton species from 2015 to 2022.

## Collection data

### Collection name

The zooplankton collections of the Department of Ecology National Research Lobachevsky State University of Nizhny Novgorod

## Usage licence

### Usage licence

Other

### IP rights notes

CC BY 4.0

## Data resources

### Data package title

Zooplankton (Rotifera, Cladocera, Copepoda) of tributaries of the Cheboksary Reservoir (Middle Volga, Russia)

### Resource link

https://doi.org/10.15468/b2ym8s

### Alternative identifiers

https://www.gbif.org/dataset/ef750680-b430-4cbb-9643-c4f49729a11c

### Number of data sets

1

### Data set 1.

#### Data set name

Zooplankton (Rotifera, Cladocera, Copepoda) of Tributaries of the Cheboksary Reservoir (Middle Volga, Russia)

#### Data format

Darwin Core

#### Download URL

http://gbif.ru:8080/ipt/archive.do?r=small-rivers-zooplankton

#### Description

The dataset provides information on 259 taxа, including 257 species and subspecies of zooplankton, as well as two genera *Bythotrephes* Leydig, 1860 and *Notommata* Ehrenberg, 1830 (rotifers – 143, cladocerans – 80, copepods – 34) from 36 families found in tributaries of the Cheboksary Reservoir and documented simultaneously with the coordinates. The families Chydoridae (35 species), Brachionidae (31) and Cyclopidae (27) were the largest in terms of species richness. The dataset has 6710 records.

In the dataset, each observation includes basic information: location (latitude and longitude), observation date, observer name and identifier. The coordinates were recorded in situ using a Garmin eTrex 32x (Garmin Ltd., USA).

**Data set 1.** 

Column label	Column description
eventID (Event core, Occurrence extension)	An identifier for the set of information associated with an event (something that occurs at a place and time).
parentEventID (Event core)	An identifier for the broader that groups this and potentially others.
waterBody (Event core)	The name of the water body in which the location occurs.
habitat (Event core)	A category or description of the habitat in which the event occurred.
decimalLatitude (Event core)	The geographic latitude of location in decimal degrees.
decimalLongitude (Event core)	The geographic longitude of location in decimal degrees.
geodeticDatum (Event core)	The ellipsoid, geodetic datum or spatial reference system (SRS), upon which the geographic coordinates given in decimalLatitude and decimalLongitude are based.
continent (Event core)	The name of the continent in which the location occurs.
country (Event core)	The name of the country in which the location occurs.
countryCode (Event core)	The standard code for the country in which the Location occurs.
stateProvince (Event core)	The name of the next smaller administrative region than country (state, province, canton, department, region etc.) in which the location occurs.
samplingProtocol (Event core)	The names of, references to, or descriptions of the methods or protocols used during an event.
year (Event core)	The integer year in which the Event occurred.
month (Event core)	The ordinal month in which the Event occurred.
day (Event core)	The integer day of the month on which the Event occurred.
sampleSizeValue (Event core)	A numeric value for a measurement of the size (time duration, length, area or volume) of a sample in a sampling Event.
sampleSizeUnit (Event core)	The unit of measurement of the size (time duration, length, area or volume) of a sample in a sampling Event.
samplingEffort (Event core)	The amount of effort expended during a Event.
eventDate (Event core)	The date when material from the trap was collected or the range of dates during which the trap collected material
coordinateUncertaintyInMetres (Event core)	The horizontal distance (in metres) from the given decimalLatitude and decimalLongitude describing the smallest circle containing the whole of the terms.
occurrenceID (Occurrence extension)	An identifier for the Occurrence (as opposed to a particular digital record of the occurrence).
scientificName (Occurrence extension)	The full scientific name including the genus name and the lowest level of taxonomic rank with the authority.
kingdom (Occurrence extension)	The full scientific name of the kingdom in which the taxon is classified.
phylum (Occurrence extension)	The full scientific name of the phylum or division in which the taxon is classified.
class (Occurrence extension)	The full scientific name of the class in which the taxon is classified.
order (Occurrence extension)	The full scientific name of the order in which the taxon is classified.
family (Occurrence extension)	The full scientific name of the family in which the taxon is classified.
individualCount (Occurrence extension)	The number of individuals present at the time of the Occurrence.
basisOfRecord (Occurrence extension)	The specific nature of the data record.
organismQuantity (Occurrence extension)	A number or enumeration value for the quantity of Organisms.
organismQuantityType (Occurrence extension)	The type of quantification system used for the quantity of organisms.
recordedBy (Occurrence extension)	A person, group or organisation responsible for recording the original Occurrence.
identifiedBy (Occurrence extension)	A list of names of people, who assigned the Taxon to the subject.
taxonRank (Occurrence extension)	The taxonomic rank of the most specific name in the scientificName.
establishmentMeans (Occurrence extension)	Statement about whether a Organism has been introduced to a given place and time through the direct or indirect activity of modern humans.

## Figures and Tables

**Table 1.** Hydrological characteristics of tributaries of the Cheboksary Reservoir (Yablokov 1972, Ryzhavskiy 1981, Gelashvili et al. 2005).

Rivers	River length, km	Basin area, km^2^	Depth, m	Lower reach, m^3^/sec	Flow velocity, m/sec
Uzola	147	1,920	0.1–0.4	NA	0.2–1.0
Chernaya (Zavolzhye)	12	49	0.1–1.0	0.3	NA
Trestyanka	17	73	0.1–0.5	NA	NA
Zhuzhla	18	78	0.1–1.0	NA	NA
Pyra	36	155	0.1–0.4	NA	NA
Chernaya (Pravoberezhye)	19	61	0.6–1.0	0.27	0.1–0.2
Linda	122	1,630	0.1–0.5	NA	NA
Vezloma	52	408	0.2–3.0	NA	NA
Oka	1,500	245,000	1.3–15.0	1,258	0.2–0.4
Kudma	144	3,220	0.4–1.2	5.75	0.1–1.2
Sundovik	97	1,120	0.1–4.0	NA	0.1–0.9
Kerzhenets	290	6,140	0.5–8.0	19.6	0.1–0.8
Sura	841	67,500	0.5–12.0	260	0.1–0.5
Vetluga	889	39,400	1.5–3.0	255	0.3–0.6

**Table 2.** Species richness by family of zooplankton in tributaries of the Cheboksary Reservoir.

Family	Number of Species	Family	Number of Species
Rotifera (total – 143)		Cladocera (total – 80)	
Asplanchnidae	5	Bosminidae	7
Brachionidae	31	Cercopagidae (genera Bythotrephes)	NA
Collothecidae	1	Chydoridae	35
Lepadellidae	8	Daphniidae	19
Conochilidae	3	Eurycercidae	2
Dicranophoridae	1	Ilyocryptidae	5
Euchlanidae	11	Leptodoridae	1
Filinidae	2	Macrotricidae	2
Flosculariidae	2	Moinidae	2
Gastropodidae	4	Ophryoxidae	1
Hexarthridae	2	Polyphemidae	1
Lecanidae	16	Sididae	5
Mytilinidae	5		
Notommatidae	12	Copepoda (total – 34)	
Philodinidae	2	Cyclopidae	27
Proalidae	2	Diaptomidae	3
Scaridiidae	1	Temoridae	4
Synchaetidae	14		
Testudinellidae	6		
Trichocercidae	12		
Trichotriidae	3		
Order Bdelloidea	NA		
